# FLDID: Federated Learning Enabled Deep Intrusion Detection in Smart Manufacturing Industries

**DOI:** 10.3390/s22228974

**Published:** 2022-11-19

**Authors:** Priyanka Verma, John G. Breslin, Donna O’Shea

**Affiliations:** 1 Data Science Institute, University of Galway, H91TK33 Galway, Ireland; 2 Department of Computer Science, Munster Technological University, T12P928 Cork, Ireland

**Keywords:** smart manufacturing, IIoT, cyber threats, Industry 4.0, federated learning, deep learning, intrusion detection

## Abstract

The rapid development in manufacturing industries due to the introduction of IIoT devices has led to the emergence of Industry 4.0 which results in an industry with intelligence, increased efficiency and reduction in the cost of manufacturing. However, the introduction of IIoT devices opens up the door for a variety of cyber threats in smart industries. The detection of cyber threats against such extensive, complex, and heterogeneous smart manufacturing industries is very challenging due to the lack of sufficient attack traces. Therefore, in this work, a Federated Learning enabled Deep Intrusion Detection framework is proposed to detect cyber threats in smart manufacturing industries. The proposed FLDID framework allows multiple smart manufacturing industries to build a collaborative model to detect threats and overcome the limited attack example problem with individual industries. Moreover, to ensure the privacy of model gradients, Paillier-based encryption is used in communication between edge devices (representative of smart industries) and the server. The deep learning-based hybrid model, which consists of a Convolutional Neural Network, Long Short Term Memory, and Multi-Layer Perceptron is used in the intrusion detection model. An exhaustive set of experiments on the publically available dataset proves the effectiveness of the proposed framework for detecting cyber threats in smart industries over the state-of-the-art approaches.

## 1. Introduction

Industry 4.0 is a new phase of the industrial revolution that heavily relies on internet-connected hardware, automation, ML, and real-time data to enable the production process with negligible human intervention. The objective of Smart Manufacturing (SM) is to distinguish openings for mechanizing activities and use data analytics to further advance manufacturing performance. SM is a precise utilization of the Industrial Internet of Things (IIoT) [1]. IIoT comprises inserting sensors in the manufacturing system to collect statistics on their functional status and performance. The amalgamation of IIoT technology in manufacturing brings adaptability and greater efficacy of production in the industrial environment [2]. However, the introduction of IIoT in the industrial manufacturing process brings new challenges such as: security, physical damage, seamless integration, and skill gap [3,4,5]. SM industries are exposed to a variety of cyber attacks such as DoS, eavesdropping, ransomware, side channel, port scan, passive replay, malicious code injection, buffer overflow and many more [6].

When comparing the effects of these attacks on traditional IT systems and manufacturing systems, the results are catastrophic for manufacturing, because in manufacturing systems damages may include physical damage to the production system, physical environment and people. In Industry 4.0, as it is hard to identify the reason for the problem, possible sets of attacks and their effects are larger in smart manufacturing systems than in traditional IT systems [7].

Thus, this drives the need for new anomaly detection approaches using AI. As AI for cybersecurity tools has improved the robustness, resilience and response to cybersecurity attacks [8,9], by watching a network, i.e., monitoring traffic and events and identifying cybersecurity threats such as malware [7], software vulnerabilities [10], fraudulent payments and network intrusion detection [11]. It does this by modeling normal (and in some cases anomalous) behaviors and classifying events using either supervised or unsupervised learning techniques and issuing alerts when anomalous behavior is observed.

### Vulnerabilities in Smart Manufacturing

To comprehend the potential attack vectors, one must first comprehend the system’s probable weaknesses. However, a lot of studies have been conducted to discover such vulnerabilities in smart manufacturing, but there will always be a new set of vulnerabilities that demand the development of advanced detection systems. Manufacturing systems are designed without considering security. It may be pre-assumed that these systems are isolated, so will not be subjected to security attacks. Hardware and software security is not considered while building these systems. As with the lack of literature and understanding related to vulnerabilities in SM, it can only be understood based on the behavior of other systems when they are attacked.

This shows that there is a high need, and also increased interest, observed in the literature to address such issues in smart manufacturing. The state of art approaches show their diversion towards using AI-based threat detection approaches in the past few years. Intrusion detection is the most commonly used method in IIoT to detect the presence of an attack so as to minimize its effects [12]. However, huge data produced by IIoT devices have become a tough task to be handled with such anomaly detection techniques. Some other examples are [13] used Q-learning to handle threats in software-defined IIoT. In [8], the author proposed a deep learning-based solution to detect attacks in smart grid CPS. Further related work on cyber threat detection in smart manufacturing is discussed under Section 2.

It is found that AI-based threat detection techniques used in SM, unfortunately, rely on the fact that there is always a large number of examples of various cyber threats available to build a high-quality intrusion detection model. However, in reality, one SM industry will have only a limited number of cyber threat examples to build the model which is quite challenging. Moreover, the owners of SM industries are not willing to share their information and data with each other to build a high-quality comprehensive model due to privacy concerns. So, in such a scenario, it is quite difficult and an intractable task to build a high-quality model to detect cyber threats in SM industries.

To address such issues, a novel distributed learning-based FL is utilized. FL enables collaborative decentralized training of an intrusion detection model [14,15,16]. In FL, the model gradients are optimized collectively by a substantial number of edge devices without disclosing their personal data. Therefore, in this work, we propose an FL-enabled deep intrusion detection framework to detect cyber threats in SM industries. In the proposed FL-enabled framework, the Deep Intrusion Detection model is used to detect cyber threats using a hybrid CNN+LSTM+MLP model. Moreover, to ensure the privacy of model gradients and to handle FL-based attacks Paillier encryption is used. The main contributions of this paper are:

1. Developed a federated learning-enabled framework to construct a comprehensive intrusion detection model which can collaboratively train the model on multiple data from different industries without disclosing it to each other. As data does not leave the premises, thus data privacy is also achieved.
2. A proposed Deep Intrusion Detection model for cyber threat detection in SM using CNN, LSTM, and MLP. The proposed model is proven to be efficient in detecting cyber threats and incorporated with the federated learning framework.
3. Proposed a Paillier-based encryption to provide secure communication throughout the training process, in order to safeguard the privacy of model gradients and to handle the threats against the federated learning framework.
4. Tests carried out on an IIoT-based dataset using the proposed FLDID framework to prove its effectiveness in the industrial environment as well.

The rest of the paper is organized as: the present approaches used for cyber threat detection are discussed in Section 2. The system model, assumptions made and threat model considered in this paper are presented in Section 3. Section 4 describes the material and methods used and Section 5 shows the evaluation of the proposed framework and discusses the observations and results. Finally, Section 6 presents the conclusion and discusses the future work.

## 2. Related Work

Recent research has shown a tremendous increase in interest in addressing the issues of cybersecurity in smart manufacturing industries. Therefore, many researchers are focusing on developing AI-based Network Intrusion Detection Systems for smart manufacturing industries. These Intrusion Detection Systems monitor and detect intrusions across the industrial network. The IDS are broadly classified into signature-based IDS and anomaly-based IDS. Anomaly-based IDS have gained major attention for intrusion detection, due to their capability for identifying novel attacks from complex and heterogeneous data generated from the IIoT devices in SM industries [17].

In the literature, there exist a wide range of anomaly detection methods proposed for cybersecurity in traditional networks [18,19,20,21]. However, these existing anomaly detection methods are not compatible with the new Industry 4.0 environment and do not achieve good accuracy. Therefore, to improve the accuracy of such systems while handling complex and heterogeneous data in SM industries, ML techniques have been employed [22]. For example, [23] investigates the problem of intrusion detection and offers a solution based on decision trees. In the paper [24], the author proposed a Gaussian Naive Bayes with LSTM model to identify two different types of abnormalities. One is hardware anomalies, particularly those produced by various types of sensors, which may have issues including interference from the environment, device malfunction, and misreadings. Another is software anomalies, which can result in aberrant or altered data gathering due to programme exceptions, transmission faults, and malicious attacks. For the purpose of detecting DDoS in IoT networks, the author [25] used a number of ML techniques and attains high detection accuracy by utilizing network behaviors for the feature selection process. Another study developed a framework for anomaly detection [26] that utilized both a temporal-spatial model and a logging-tracing model to identify anomalies in network traffic.

Deep learning methods have recently produced impressive results and emerged as one of the successful ways for intrusion detection systems. A variety of deep learning approaches to security monitoring were reviewed by the author [27]. They contrasted common deep learning models with a few traditional ML techniques. The findings in the review showed how useful deep learning techniques are for protecting against cybercrime. To specifically detect intrusions, the author [28] incorporated deep neural networks and used the KDD99 dataset to prove the efficacy of the proposed approach. A dense random neural network was constructed in another study [29] to identify cyberattacks. In contrast, in [30], the author discovered that a deep neural network with three hidden layers produced classification results that were superior to those achieved with more classes when fewer invasive classes were used.

All of these centralized learning techniques necessitate central storage (server) of all data acquired on local devices. This necessity not only increases concerns about threats pertaining to privacy and data leaks, although it also heavily requires the server’s storing and computational capacities when the data is large. However, the distributed approach allows multiple systems to train model replicas with different data groups in parallel to solve the above problem. However, it still requires access to the entire training data set in order to partition it into uniformly distributed shards, thereby causing security and privacy issues.

To overcome such a problem FL has appeared as an intriguing method. FL intends to develop a global model that can be trained on data spread across several devices while maintaining data privacy. Some of the research using FL are: In [31], the authors proposed a DNN-based client-server FL architecture. A large part of the suggested models achieved pretty good accuracy (99.00%) came from the dataset utilized, N-BaIoT, which is regarded as being highly simple and straightforward. However, adversarial attacks such as data and model poisoning dramatically reduced performance, demonstrating the requirement for more effective defenses. An FL-enabled GRU model is proposed in [32] which employed a GRU to find the Mirai attack in an IoT network. However, the proposed model successfully detects the Mirai attack but lacks resilience and robustness while aggregating the gradients at the server. In the same year, an MT-DNN-FL has been proposed in [33] to carry out the work for identifying anomalies in the network. An FDAGMM was given in [34] to address the classic DAGMM’s performance issue for network anomaly detection due to a lack of data.

The author in [35] used the X-IIOTID dataset to prove the effectiveness of their proposed approach for detecting targeted ransomware attacks for IIoT. They used asynchronous peer-to-peer FL and DL algorithms to detect ransomware attacks. The result shows that the proposed approach achieves a detection accuracy of 98.33%. Similarly in [36] author developed safe data sharing architecture for IIoT devices using FL to handle the data breach and achieved an accuracy of 99.79% accuracy over the X-IIoTID dataset.

Despite the efficient anomaly identification achieved by earlier investigations, the following issues still exist. The first open issue is how to provide privacy-preserving intrusion detection. Second, given that clients and servers may be partially honest and how anomaly detection accuracy is improved. Third, the existing techniques are tested on an intrusion dataset created on a traditional network. However, it completely differs from the SM environment as it lacks interoperability and heterogeneity. Thus, in this paper, the FL-enabled deep intrusion detection framework is proposed and tested on the IIoT-based “X-IIoTID” intrusion dataset [37] to solve the above problems in smart manufacturing systems.

## 3. System Model, Assumptions and Threat Model

### 3.1. System Model

**Figure 1:**
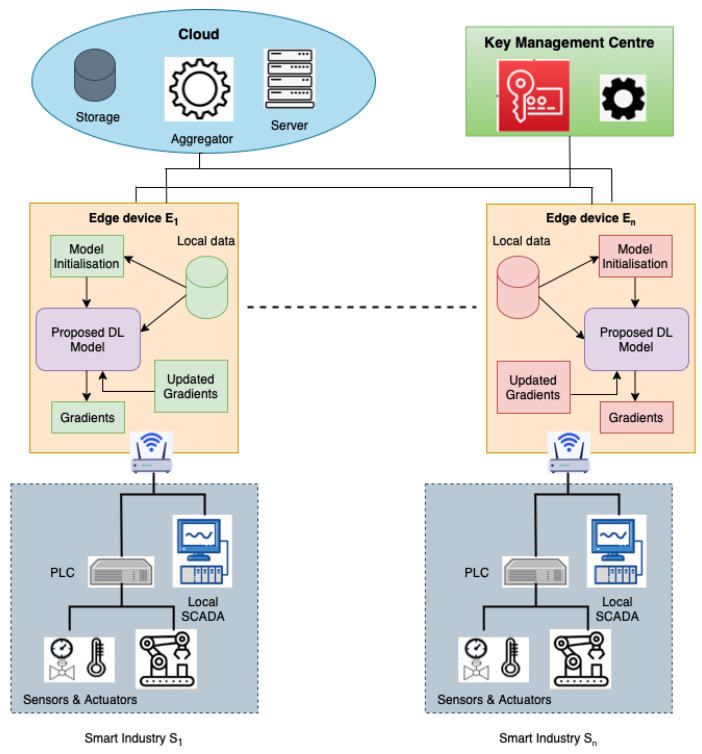
System architecture for proposed framework.

The system model used in this paper is presented in Figure 1. The proposed FLDID framework consists of edge devices as representative of SM industries, cloud servers/aggregators, and key management centers.

1. Edge Devices: Each edge device is representative of SM industries taking part in the process of collaborative learning. These devices have the local data collected from its associated SM industry and are responsible for building the local DID model using this data. It is also responsible for communicating with the cloud server to send local model parameters, receive aggregated model parameters from the cloud server, and again perform the training on the received model. These steps are recursively performed until convergence. The local model is placed on the edge devices which are capable of performing intrusion detection on their local data. In this work, we used a CNN+LSTM+MLP-based DL model for intrusion detection.
2. Cloud Server: It is accountable for collaboratively building the DID model in a federated way. The cloud server consists of two major functionalities: (a) initialization of global model parameters and sharing it with local edge devices, (b) aggregating the parameters uploaded by edge devices until the model converges and sends it back to the edge devices.
3. Key Management Centre: The KMC is responsible for ensuring secure communication between the cloud server and the edge devices. It establishes a secure channel between them using a Paillier-based cryptosystem. The Paillier cryptosystem is a partially homomorphic encryption scheme that allows operations on encrypted data. KMC is also responsible for generating public and private keys used in the Paillier cryptosystem.

### 3.2. Assumptions

The major assumptions considered in this work are:

- Cloud server is considered a trustworthy party but curious, which is honestly performing its assigned task but quite interested in knowing the model gradients.
- KMC is assumed to be the fully honest party ensuring secure communication between edge devices and the cloud server.
- Edge devices are considered to be partially trustworthy, as they follow the process but may be curious about the other edge device’s data resources.

### 3.3. Threat Model

In this work, we considered the cyber threats launched against SM industries along with the threats targeting the federated learning framework. The major threats launched against SM industries are DoS, DDoS, Reconnaissance, Exploitation, Weaponization, RDoS, Crypto Ransomware, Command/Response Injection attacks and many more. We considered all the mentioned cyber threats in one category as attacks for this work.

The threats considered against the FL framework in this work are membership inference attacks, unintentional data leakage and reconstruction through inference, and GANs-based inference attacks.

## 4. Materials and Methods

This section presents the elaboration of the proposed FLDID framework by first outlining the overall workflow of the framework, followed by introducing the Deep Intrusion Detection model designed for intrusion detection, and then discussing the Paillier encryption scheme used for securing the communication between edge devices and the server.

Algorithm 1 presents the complete procedure of the proposed FLDID framework. The performance of deep learning models can be enhanced by collaboratively training the model on multiple data resources which consist of a variety of attack examples. Thus, the proposed FLDID framework is designed to allow collaborative learning between different SM industries and to build a robust and comprehensive intrusion detection model using federated learning with secure communication. The major phases of the proposed FLDID framework are described below:
**Algorithm 1** Secured FL procedure**Input:** Set of Edge devices $E_{n}$ and their associated local data $D_{n}|n\in N$, No. of communication rounds *R***Output:** Global DID model  1: Cloud server initialize model parameter $W_{0}$ and η,B,ζ,ρ,τ  2: Each $E_{n}$ informs the data size $|D_{n}|$ to server;  3: Server computes contribution ratio $\alpha_{n}=|D_{n}\left|/(\right|D_{1}\left|+\right|D_{2}\left|+\ldots +\right|D_{N}\left|\right)$;  4: For each $E_{n}$ KMC generates key pair (PubK,PriK) using KeyGen(n) and establishes secure channel between each edge device and server;  5: For r = 1 to R;   (i) $E_{n}$ computes $r^{th}$ round model gradients $W_{n}^{r}$ using Algorithm 2;   (ii) Encrypt model gradients $W_{n}^{r}$ as $E\left(W_{n}^{r}\right)=Encrypt\_grad\left(W_{n}^{r},PubK\right)$;   (iii) Upload $E(W_{n}^{r})$ to cloud server;   End  6: Cloud server aggregates $E(W_{n}^{r})$ as $C=Aggr\_grad(E\left(W_{1}^{r}\right),\left(W_{2}^{r}\right),\ldots \alpha_{1},\ldots \alpha_{n})$;  7: Server distributes *C* to all edge devices $E_{n}$  8: To obtain new global model $E_{n}$ performs decryption as $\tilde{W^{r}}=Decrypt\_grad\left(C,PriK\right)$  9: $E_{n}$ updates model gradients using $\tilde{W^{r}}$;  10: r←r+1;

- **Step 1 (Model initialization):** In this step, the server selects and sends the initial parameters $W_{0}$ for the DID model and other necessary parameters required for model training such as: Batch size *B*, Learning rate η, loss function ζ, momentum ρ, and decay τ to the edge devices. Moreover, each edge device $E_{n}$ associated with SM industries $S_{n}$ informs the server about the size of the data $D_{n}$ it has from the industry, where n∈N=1,2,…,N, this helps the cloud server to calculate the contribution ratio $\alpha_{n}$ for each edge device. A positive integer *R* defines the number of communication rounds between the edge devices and the cloud server.
- **Step 2 (Key generation):** In addition to the above parameters, next the public key PubK and the private key PriK are generated using KeyGen(n) by the KMC which is used in Paillier encryption to establish a secure path between the server and edge device.
- **Step 3 (Local model training):** Based on the initial model parameters $W_{0}$, η,B, ζ, ρ, τ received from the cloud server, each edge device performs the model training on their local data $D_{n}$. The model used for training at the edge devices is described in Section 4.1. In the proposed DID model, a hybrid CNN+LSTM+MLP model is used to train the model for detecting intrusions in smart manufacturing. The elaborated training process is presented in Algorithm 2.
- **Step 4 (Gradient encryption):** After training the model on the local data each edge device $E_{n}$ encrypts the model gradients $W_{n}^{r}$ using $Encrypt\_grad(W_{n}^{r},PubK)$, where $W_{n}^{r}$ are the model gradients after training at edge device $E_{n}$ in the $r^{th}$ round. Then, the encrypted gradients $E(W_{n}^{r})$ of the local models by each edge device are sent to the cloud for aggregation to generate the comprehensive global model.
- **Step 5 (Global model construction):** Cloud server aggregates the encrypted model gradients received from each edge device participating in the process of collaborative learning. The gradients are aggregated using $Aggr\_grad(E\left(W_{1}^{r}\right),E\left(W_{2}^{r}\right),$$\ldots E(W_{n}^{r}),$$\alpha_{1},\ldots \alpha_{n})$, where $\alpha_{n}$ is contribution ratio of each edge device. Then the aggregated encrypted gradients are sent back to edge devices as a cipher text *C*.
- **Step 6 (Local model updation):** At each edge device after receiving the global model (aggregated gradients) as a cipher text, each edge device performs decryption Decrypt_grad(C,PriK) using the private key. After receiving the decrypted gradients $\tilde{W^{r}}$, local DID models are then updated and retrained with their local data.

**Algorithm 2** DL model training**Input:** 
$W_{0},\eta ,B,\zeta ,\rho ,\tau$**Output:** 
$W_{n}^{r}$  1: Divide $D_{n}$, into equal size *B* batches with feature vector *x*;  2: Set $W_{n}^{r}$ with initial values;  3: For each Batch;    $c_{1}\leftarrow$ Forward *x* to $Conv_{1}$;    $c_{2}\leftarrow$ Forward $c_{1}$ to $Conv_{2}$;    λ← Flatten $\left(c_{2}\right)$;    $H^{\prime }\leftarrow$ Forward λ to $LSTM_{1}$;    μ← Forward $H^{\prime }$ to $LSTM_{2}$;    M← Forward μ to Dense;    γ← Dropout (M);    ν← Forward γ to Output(Sigmoid);  4: Compute loss function using:    $\zeta =-\frac{1}{B}\sum_{i=0}^{1}x_{i}.log{\hat{x}}_{i}+\left(1-x_{i}\right).log\left(1-{\hat{x}}_{i}\right)$;  5: Update $W_{n}^{r}$;  6: Repeat until ζ converges;

### 4.1. Proposed Deep Intrusion Detection Model

**Figure 2:**
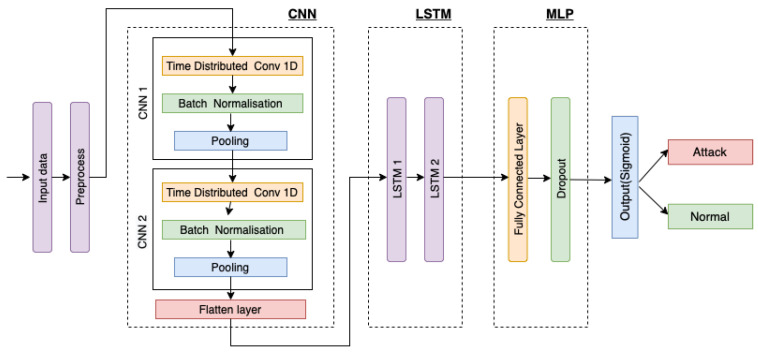
Architecture of designed DL model.

This section presents the hybrid deep learning-based intrusion detection model. The designed DID model consists of the pre-processing unit, and a CNN and LSTM unit followed by an MLP unit as shown in Figure 2.

The approach followed provides a diverse combination of convolutional and recurrent neural networks. As network traffic events follow time series patterns, it is possible to categorize the present network connection record using the traffic connection records from the past. We feed freshly produced features from CNN’s unit to LSTM and sequentially to MLP in order to capture the time series patterns over time-steps and obtain the desired result.

The proposed DID model was selected following rigorous experimentation of several DL techniques, including CNN, MLP, and LSTM with different numbers of layers, as well as their topologies, network parameters, network architecture and hybrid combinations. CNN and its variations in our experimental setup and dataset outperforms traditional ML classifiers on comparing with them. The capacity of CNN to extract high-level feature representations, which represent the abstract form of low-level feature sets of network traffic links, is the fundamental driver for this success. The combination of two-layer CNN, two LSTM layers, and a densely integrated MLP layer produced the best results on the dataset considered in this work. Therefore, we decided to incorporate this model as an intrusion detection model into our proposed federated learning architecture to detect cyber threats in smart manufacturing. Each unit of the proposed DID model is described below:

#### 4.1.1. Pre-Processing Unit

First, the data is pre-processed and given to the input layer. Data pre-processing is an important step as all the ML algorithms deal with numerical data. So the features that are of string types need to be label encoded. The numerical data that is obtained after the label encoding does not correspond to the same range. So, to normalize all the feature values, the min-max normalization technique is used [38]. This makes all the feature values come under a similar range. The minimum value of the feature transforms into 0, the maximum value transforms into 1, and every other value gets transformed between 0 and 1. So, the final range of the values of the feature is in between [0, 1].

#### 4.1.2. CNN Unit

CNN is employed in the proposed DID model to extract fine-grained characteristics. Two (1-D) CNN layers constitute this unit. Each layer constitutes convolution, batch normalization, and pooling layers. CNN’s local perception and weight sharing can enhance model learning by significantly reducing the number of parameters. These modules build layered structures that increasingly draw out more precise information by stacking convolutional layers and achieve sampling aggregation by employing pooling layers. Each convolution layer comprises convolution kernels. The layer of batch normalization enables the network’s layers to learn more independently. The output of the earlier layers is normalized using it. Batch normalization helps in high-yield learning and also prevents the over-fitting of the model. It serves as a bridge between the pooling layer and the convolution layer. As a result of this unit, a sequence of *m* features with size (n∗m) is achieved, where *n* specifies the length.

Following the convolution operation of the convolution layer and batch normalization layer, the features received possess high dimensionality. Thus, to reduce the feature dimension and training cost pooling layer is also employed. Given one-dimensional data from the time series events collected from SM industries as input vector $x=(x_{1},x_{2},\ldots x_{n},l)$, where ($x_{n}\in R^{d}$ is the features available and l∈R is the class label) being the input of the designed model, first given to the CNN unit. In this unit, a feature map $z_{map}$ is constructed by the 1D convolution layer using convolution operation with filter $\omega \in R^{zd}$. From the available features *z*, a new feature map $z_{map}$ is obtained as:

(1) $$\theta_{j}^{z_{map}}=tanh\left(\omega^{z_{map}}x_{j:j+z-1}+a\right)$$

where a∈R denotes the bias, filter θ is applied to each feature set *z* to obtain the feature map as

(2) $$\theta =[\theta_{1},\theta_{2},\ldots ,\theta_{n-z+1}]$$

further, this output is sent to the batch normalization and pooling layer. The complete process of the CNN unit is represented as

(3) $$c_{1}=Conv_{1}\left(x\right)$$

(4) $$c_{2}=Conv_{2}\left(c_{1}\right)$$

(5) $$\lambda =Flatten(c_{2})$$

where $Conv_{1}$ and $Conv_{2}$ represent the convolutional blocks with batch normalization and pooling operation in the CNN and $c_{1},c_{2}$ are the hidden vectors. Further, the output of the second convolution block is flattened using the flatten layer and λ is the final output of the CNN units. λ is the *m* feature sequence of length *n* and can be represented as (n∗m).

#### 4.1.3. LSTM Unit

The output of CNN is used as input to LSTM in the envisaged DID module. LSTM is the superior version of Recurrent Neural Network (RNN). Instead of using standard simple RNN units, LSTM uses memory blocks to handle the issue of disappearing and shattering gradients. LSTM manages long-term dependencies far more easily than conventional RNNs. This shows that LSTMs are capable of remembering and connecting knowledge that is far older than the present [39]. A memory block in an LSTM is a relatively sophisticated processing unit made up of one or more memory cells. LSTM uses gates such as forge gates $F_{T}$, input gates $I_{T}$, and output gates $O_{T}$. Two multiplicative gates make up the input and output gates. The LSTM gate structures are defined as:

(6) $$F_{T}=\varphi \left(W_{F}\cdot \left[H_{T-1},X_{T}\right]+b_{F}\right),$$

(7) $$I_{T}=\varphi (W_{I}\cdot \left[\left[H_{T-1},X_{T}\right]+b_{I}\right),$$

(8) $${\tilde{S}}_{T}=tanh(W_{C}\cdot \left[\left[H_{T-1},X_{T}\right]+b_{C}\right),$$

(9) $$S_{T}=F_{T}*S_{T-1}+I_{T}*{\tilde{S}}_{T},$$

(10) $$O_{T}=\varphi (W_{O}\cdot \left[\left[H_{T-1},X_{T}\right]+b_{O}\right),$$

(11) $$H_{T}=O_{T}*tanh\left(C_{T}\right),$$

where $W_{F},W_{I},W_{C},W_{O},$ and $b_{F},b_{I},b_{C},b_{O}$ are the weight metrics and bias vectors for input $X_{T}$ at time step *T*. ∗ represents the element-wise multiplication, $S_{T}$ represents the cell state. $H_{T-1}$ and $H_{T}$ are the hidden layer states at step T−1 and at *T*, respectively. φ is the activation function used.

In this study, LSTM is designed to aid precise prediction in the time series data to find anomalies. LSTM processes λ (output of CNN unit) in the following way:

(12) $$H^{\prime }=LSTM_{1}\left(\lambda \right),$$

(13) $$\mu =LSTM_{2}\left(H^{\prime }\right)$$

#### 4.1.4. MLP Unit

The MLP module is composed of a dense layer and an output layer separated by a dropout layer. In order to avoid model over-fitting, the output of the LSTM μ in this module is provided as input to an MLP unit with dropout as follows:

(14) M=Dense(μ)

(15) γ=Dropout(M)

(16) ν=Output(Sigmoid(γ))

where Dense and Output are the two fully connected layers, Dropout represents the dropout layer. *M* and γ are the output of dense layer 1 and the dropout layer, respectively. Additionally, because the output predicted class contains two values, either attack or normal, the output of the MLP dropout layer is passed to the output layer with the sigmoid function.

We utilized binary cross entropy as the loss function since the proposed DID model uses binary classification to identify attack and non-attack traces in the smart manufacturing industries, which is defined as follows:

(17) $$\zeta =-\frac{1}{B}\sum_{i=0}^{1}x_{i}.log{\hat{x}}_{i}+\left(1-x_{i}\right).log\left(1-{\hat{x}}_{i}\right)$$

where *B* is the batch size, $x_{i}$ is the corresponding target value and ${\hat{x}}_{i}$ is the corresponding predicted value.

Algorithm 2 shows the steps involved in training the local models at each edge device. Each edge device $E_{n}$ trains the local model on their local data $D_{n}$. On each communication round, upon receiving cipher text *C*, each edge device decrypts it and obtains new gradients $\tilde{W^{r}}$ and updates $W^{r}$ accordingly. After updation, the DID model is retrained at each edge device $E_{n}$ on their local data $D_{n}$ until model convergence.

### 4.2. Encryption Method for Secure Communication

In order to ensure the privacy of data and model gradients, many encryption methods have been developed. Although, none of the methods are capable of performing computations on the encrypted data. Thus, to handle such issues and in order to mitigate malicious eavesdroppers and other attacks against the FL framework as mentioned in Section 3.3, the Paillier-based encryption method is used. It is a type of partial homomorphic encryption scheme which is capable of performing computations of encrypted data.

In this encryption scheme, the key management center is only responsible for the generation of public and private keys for the clients but has no access to the data. KMC does not even have any idea about what data is being encrypted by the clients.

In the case of the server, the data received here is in the form of cipher text which is encrypted by the clients. Moreover, the operations performed at the server are all homomorphic operations which are performed without the decryption process. So even if the server is compromised no plain text data is obtained, because at the server all the data is in encrypted form.

From clients, they obtain the key pair from KMC, then encrypt the gradients and send it to the server for aggregation. The server sends back the global model but in encrypted form. During the entire process, clients cannot access each other’s data or the gradients which can ensure data security.

This process is majorly performed in three steps:

1. KeyGen: The key management center generates the Public key PubK=(p,q) and Private key PriK=(δ,κ) using Paillier cryptosystem as mentioned in [40].
2. Encrypt_grad: Here, the model gradients $W_{n}^{r}$ are encrypted using PubK(p,q) and results in $E(W_{n}^{r})$. For example, if *m* is the plain text and *C* is the cipher text the encryption is represented as:
  (18) $$C=q^{m}.\;r^{p}mod\;p^{2}$$
  where *r* is random number such that r<p.
3. Decrypt_grad: Each edge device performs decryption upon receiving the cipher text C from the cloud server and retrieves the updated gradients $\tilde{W^{r}}$. The decryption is performed on *C* using the private key PriK(δ,κ) to obtain the plaintext *m*.
  (19) $$m=\frac{L(C^{\delta }\;mod\;p^{2})}{\left(q^{\delta }\;mod\;p^{2}\right)}mod\;p$$

#### Analysis of Encryption Scheme

As far as the overhead and complexity of the encryption scheme are considered, each edge device $E_{n}$ is required to perform the encryption and decryption task and it can be denoted by:

(20) Encrypt_TC=O(N∗R∗E(Paillier))

(21) Decrypt_TC=O(N∗R∗D(Paillier))

where *R* is the number of communication rounds and *N* is the number of edge devices participating in the process of federated learning. D(Paillier), and E(Paillier) is the time complexity of the Paillier method, which requires total G exponentiation operation and relatively negligible multiplication operation in each round. Therefore, we can say that the computational cost at each edge device is linearly proportional to the number of gradients in the DL model. However, for the cloud server it only needs to perform *X* time of additive operation while aggregating the encrypted gradients.

To obtain the overhead of Paillier encryption, the proposed scheme is evaluated for the percentage of CPU consumption, memory occupied, and time taken to build the model. From Table 1, it is evident that the proposed framework with Paillier encryption results in increased CPU and memory consumption with high training time in comparison to the proposed framework without Paillier encryption. This shows that using the Paillier encryption adds an extra overhead to the proposed framework.

## 5. Results and Discussion

### 5.1. Environmental Setup and Parameters

The proposed federated learning framework was implemented in Python using TensorFlow (TensorFlow is an open-source library used in Python for deep learning applications) and the designed deep learning model is implemented using Keras (https://keras.io/api/, accessed on 20 July 2022) API. The proposed framework is implemented and evaluated using Python 3.0 on a MacBook pro having an Apple M1 Pro chip with 10-core CPU and 16-core GPU, 16 GB RAM, and 1TB SSD. The parameters used for the FL model and DID model based on the CNN + LSTM + MLP are described in Table 2.

### 5.2. Dataset Description

Analysis of the proposed framework was performed using the X-IIoTID dataset. X-IIoT is an IIoT-based intrusion dataset that captures the heterogeneity of network traffic as well as system activities produced by diverse IIoT devices. The features in the X-IIoTID dataset are gathered and created from a variety of data sources. The dataset has 68 attributes, including features linked to hosts and network traffic. It covers nine different attack types, including reconnaissance, weaponization, lateral movement, Command and Control (C&C), tampering, Ransom Denial of Service (RDoS), exfiltration, and crypto-ransomware. However, in this work, we are interested in classifying attacks and normal requests. As a result, we grouped them all under the heading “attack”. Classification into numerous attack categories will also be taken into consideration in future development. The X-IIoTID dataset has 820,834 cases with 68 features [37] and two labels (421,417 as normal and 399,417 as attacks). In this work, the data resource is split, with 80% of it being used for training and 20% for testing. The training portion is then further divided into equal portions for each industrial agent, which is used by them to train their local model. Keeping in mind the practical application of the federated framework, a scaling factor (or weightage) is implemented to incorporate the factor of variable data strength offered by different clients, i.e., it takes into account that the data at all the client’s end would not be in equal portions.

### 5.3. Performance Metrics Used

The metrics used to assess the proposed FLDID framework are classification Accuracy, Precision, Recall, and F1-Score. True Positive (TP), True Negative (TN), False Positive (FP), and False Negative (FN), are used to express these metrics, respectively, where:

TP: Specify the count of attack requests rightly predicted as attack;
TN: Specify the count of benign samples rightly predicted as benign;
FP: Count of benign requests falsely predicted as attack;
FN: Count of attack requests falsely predicted as benign.

1. **Accuracy:** It is the percentage of the right prediction of attack and benign requests
  (22) $$\mathrm{Accuracy}=\frac{\left(\mathrm{TP}+\mathrm{TN}\right)}{\left(\mathrm{TP}+\mathrm{TN}+\mathrm{FP}+\mathrm{FN}\right)}$$
2. **Recall:** It is defined as the ratio of right prediction of attack to the all observations in actual class
  (23) $$\mathrm{Recall}=\frac{\mathrm{TP}}{\left(\mathrm{TP}+\mathrm{FN}\right)}$$
3. **Precision:** It is the ratio of right prediction of benign request to the entire predicted benign requests.
  (24) $$\mathrm{Precision}=\frac{\mathrm{TP}}{\left(\mathrm{TP}+\mathrm{FP}\right)}$$
4. **F1-Score:** This score is the measure of test’s accuracy and calculated using Precision and Recall.
  (25) $$F1-\mathrm{Score}=2*(\frac{\left(\mathrm{Recall}*\mathrm{Precision}\right)}{\left(\mathrm{Recall}+\mathrm{Precision}\right)})$$

### 5.4. Result Evaluation

To evaluate the performance of the proposed FLDID framework, experiments are conducted under four scenarios in which the number of edge devices considered is *N* = 2, 5, 10, and 15, respectively. For each scenario, the number of rounds considered is *R* = 2, 4, 6, 8, and 10. Table 3 shows the obtained results of the proposed FLDID framework for the metrics defined in Section 5.3.

For all the scenarios, it is seen that the FLDID framework performs well on all the metrics. It is worth noting that with the proposed framework for the different number of edge devices, we achieved good accuracy, precision, recall and F-Score in all the scenarios.

**Figure 3:**
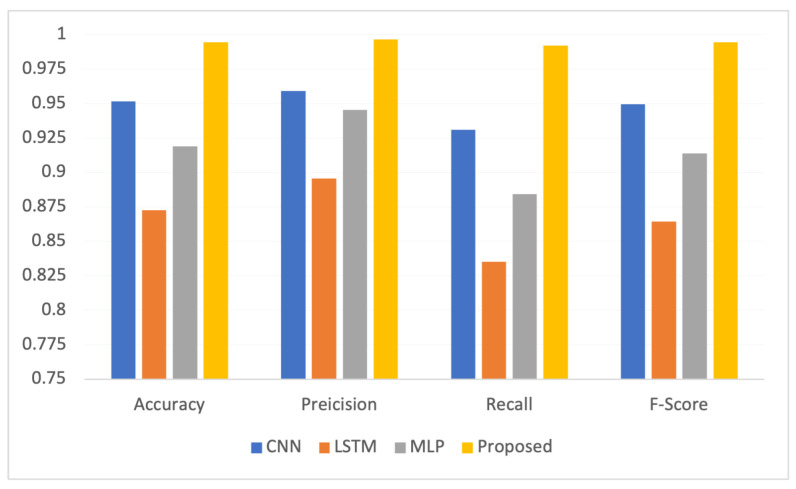
Comparison of proposed model with individual DL models.

The proposed FLDID framework is also compared with individual CNN, LSTM, and MLP models in a federated way to show the domination of the proposed model in comparison to individual DL models. Figure 3 shows that the proposed model overperforms on all the matrices in comparison to the individual models.

**Figure 4:**
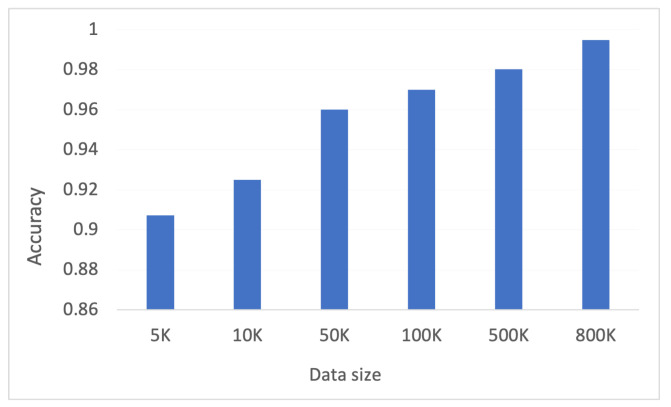
Model accuracy with different data sizes.

Moreover, to show the effectiveness of the proposed approach, the results are also evaluated on different data sizes. As shown in Figure 4, it is observed that with less data (insufficient traces) model does not achieve good accuracy, but with the increased data size (sufficient traces) good accuracy can be achieved. Therefore, federated learning is used in this work to overcome the problem of limited data for training the model and ensuring data privacy as well.

#### 5.4.1. Performance Comparison with Centralized and Isolated Models

In this section, the FLDID is compared with the central and isolated model. In a centrally built ideal model, the central entity uses all the data to build the model. While with the isolated model, the model is built at each edge device with limited data available to them. While evaluating the centralized model, it is assumed that all the smart industries allow their data to be used by the central entity to build the model. However, in isolated models smart industries do not share their data, they build the model with the limited data available to them.

**Figure 5:**
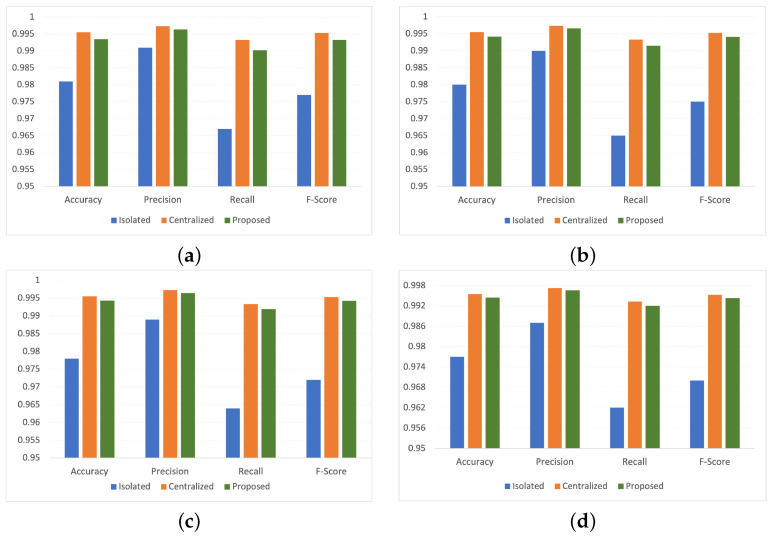
Performance comparison of FLDID with centralized and isolated models with different numbers of edge devices for *R* = 10: (**a**) N = 2, (**b**) N = 5, (**c**) N = 10, (**d**) N = 15.

Figure 5 shows the performance of the proposed, centralized and isolated model for different metrics under different scenarios for 10 rounds. As we can observe that the local models do not perform satisfactorily in comparison to the proposed models in all the scenarios due to limited data availability while training the model. Moreover, it can be seen that the proposed model gives good results and almost performs similarly to the centralized (ideal) model. However, with centralized model data privacy cannot be achieved as all the smart industries have to provide their data to the central entity for model building. However, with the proposed framework similar performance for intrusion detection can be achieved while preserving data privacy. However, with federated learning sometimes trustworthy but curious clients and servers may cause data inference attacks. As the model gradients are sent from clients to servers, which can be used in the inference attack. Thus, to ensure the privacy of model gradients and save the data from inference attacks, Paillier-based encryption is also used. However, using encryption increase the overhead of the proposed framework.

#### 5.4.2. Performance Comparison with Baseline Studies

In this section, we present the comparison of the proposed FLDID framework with some state-of-the-art studies that use the federated learning framework. The existing work considered for comparison are: (1) in the work [41] author used a simple MLP-based FL framework for intrusion detection in the health care domain; (2) in [32], GRU-based FL framework for intrusion detection in IIoT domain is used; (3) in [42], the author presents a CNN-based FL framework for the request classification. These baseline studies are re-implemented in our experimental setup and compared with the proposed FLDID framework on the X-IIoTID dataset.

**Figure 6:**
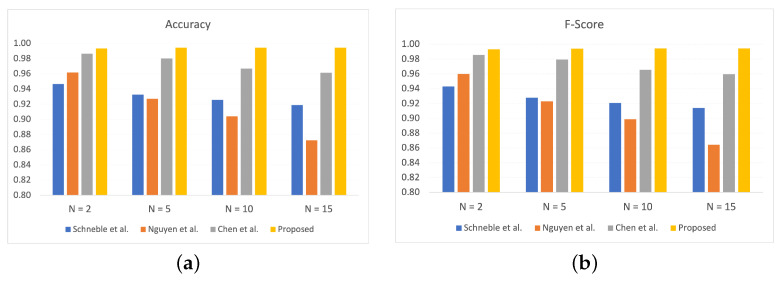
Performance comparison of FLDID with state-of-the-art approaches with different number of edge devices for *R* = 10: (**a**) N = 2, (**b**) N = 5.

Figure 6 shows the performance comparison for accuracy and F-score of FLDID with the baseline studies for the different number of edge devices, N = 2, 5, 10, 15. It is worth noting that the proposed FLDID framework outperforms all the baseline studies and achieved the highest accuracy and F-Score.

#### 5.4.3. Performance Comparison of Proposed Model with ML Classifiers

The proposed FLDID framework is also compared with ML models. The ML models considered are Support Vector Machine (SVM), K-Nearest Neighbor (KNN), Logistic Regression (LR), and Decision Tree (DT). On comparing as mentioned in Table 4, it is observed that the proposed FLDID framework and DT outperforms SVM, LR, and KNN classifiers and achieves good accuracy which is more than 99%. Moreover, KNN, DT, LR and the proposed framework consume fewer resources compared to SVM. However, as we are using a hybrid of CNN+LSTM+MLP-based deep learning model with federated learning to increase the detection accuracy, thus it results in high training time in comparison to LR, KNN, and DT. Although DT and KNN perform almost similarly to the proposed framework, they fail to ensure data privacy between different industrial agents while training the model. However, the proposed framework wins over in this context and ensures data privacy along with high accuracy which is the major concern in our scenario.

## 6. Conclusions

This work proposes a federated learning-enabled deep intrusion detection framework to detect cyber threats in smart manufacturing industries. The proposed framework enables multiple smart manufacturing industries to collaboratively build a comprehensive intrusion detection model through their representative edge devices. Moreover, in this work, the deep learning-based DID model using hybrid CNN+LSTM+MLP is used for intrusion detection. The experimental results show that the proposed FLDID framework outperforms the other state-of-the-art approaches and achieves good results for detecting cyber threats in smart manufacturing industries. While building the model collaboratively, the proposed framework also ensures it is built in a privacy-preserving way. The privacy of model parameters is achieved by building a secure channel between the edge devices and the cloud server using partial homomorphic encryption, i.e., the Paillier encryption method. However, homomorphic encryption will unavoidably result in increased overhead for the encryption and decryption process, which will substantially limit the effectiveness of training.

Therefore, in future work, the performance of the learning process will be accelerated by using efficient homomorphic encryption methods which results in low overhead. Moreover, a more robust and personalized federated learning should be considered, which helps to divide similar clients into clusters and handles the data poisoning attack. Additionally, to identify different types of attacks, we concentrate on providing the multiclass classification framework.

## Figures and Tables

**Table 1:** Comparison of proposed framework with and without Paillier encryption.

Proposed Framework	CPU	Memory	Time (in s)
Without Paillier encryption	20%	72%	3042
With Paillier encryption	83%	88%	76,920

**Table 2:** Parameters used in the proposed model.

FL Model	Learning rate (0.01), Momentum (0.9), decay (0.01), loss function (binary cross-entropy), epoch (10), number of clients (K = 2, 5, 10, 15)
DID Model (CNN + LSTM + MLP)	No. of hidden layers (11), dropout rate (0.2), CNN Layer 1 (filters (128), kernel size (3), activation function (relu)), CNN Layer 1 (filters (64), kernel size (3), activation function (relu)), pooing size (2), strides (2), LSTM layers (perceptron (50), activation function (tanh)), MLP layer (perceptron (100), activation function (tanh), output layer activation function (sigmoid)), optimizer (adam), loss (binary cross entropy)

**Table 3:** Performance of FLDID framework under 4 different scenarios.

N	R	Accuracy	Precision	Recall	F-Score
2	2	0.99032	0.99555	0.98449	0.98999
	4	0.99183	0.9946	0.98857	0.99157
	6	0.99259	0.9947	0.99004	0.99237
	8	0.99306	0.99368	0.99204	0.99286
	10	0.99348	0.99634	0.99022	0.99327
5	2	0.99373	0.99573	0.99135	0.99353
	4	0.99409	0.99621	0.99161	0.9939
	6	0.99415	0.99577	0.99219	0.99398
	8	0.99415	0.99626	0.99169	0.99397
	10	0.99428	0.99665	0.99157	0.9941
10	2	0.99432	0.99645	0.99186	0.99415
	4	0.99434	0.99637	0.99198	0.99417
	6	0.99437	0.99642	0.99198	0.99419
	8	0.99443	0.99654	0.99199	0.99426
	10	0.99438	0.99645	0.99198	0.99421
15	2	0.99441	0.99654	0.99195	0.99424
	4	0.99443	0.99644	0.9921	0.99426
	6	0.99443	0.99667	0.99185	0.99426
	8	0.99445	0.99657	0.99199	0.99428
	10	0.99447	0.99659	0.99203	0.9943

**Table 4:** Performance comparison of proposed model with other ML models.

Classifier	Accuracy	CPU	Memory	Time (in s)
SVM	0.9227	35%	90%	4265
LR	0.9148	15%	80%	36.411
KNN	0.9847	14%	75%	0.6592
DT	0.9904	19%	78%	4.975
Proposed	0.9979	20%	72%	3042

## Data Availability

The data used for this study are publicly available and can be downloaded at https://www.kaggle.com/datasets/munaalhawawreh/xiiotid-iiot-intrusion-dataset (accessed on 20 July 2022).

## Abbreviations

The following abbreviations are used in this manuscript:

AI	Artificial Intelligence
CNN	Convolutional Neural Network
CPS	Cyber Physical Systems
C&C	Command and Control
DDoS	Distributed Denial of Service
DID	Deep Intrusion Detection
DL	Deep Learning
DoS	Denial of Service
FDAGMM	Federated Deep Auto encoding Gaussian Mixture Model
FL	Federated Learning
FLDID	Federated Learning enabled Deep Intrusion Detection
FN	False Negative
FP	False Positive
GRU	Gated Recurrent Unit
IDS	Intrusion Detection Systems
IIoT	Industrial Internet of Things
KMC	Key Management Centre
LSTM	Long Short Term Memory
ML	Machine Learning
MLP	Multi Layer Perceptron
MT-DNN-FL	Multi-Task Deep Neural Network in Federated Learning
RDoS	Ransom DoS
RNN	Recurrent Neural Network
TN	True Negative
TP	True Positive
SM	Smart Manufacturing
